# Integrated Metabolomics Study of the Milk of Heat-stressed Lactating Dairy Cows

**DOI:** 10.1038/srep24208

**Published:** 2016-04-06

**Authors:** He Tian, Nan Zheng, Weiyu Wang, Jianbo Cheng, Songli Li, Yangdong Zhang, Jiaqi Wang

**Affiliations:** 1 Institute of Animal Science, Chinese Academy of Agricultural Sciences, Beijing, 100193, P.R. China; 2 The High School affiliated to Renmin University of China, Beijing, 100080, P.R. China; 3 College of Animal Science and Technology, Anhui Agricultural University, Hefei, 230036, P.R. China

## Abstract

Heat stress (HS) damages the global dairy industry by reducing milk yields and quality, harming health, and damaging the reproduction of dairy cows, causing huge economic losses each year. However, an understanding of the physiological mechanism of HS lactating dairy cows remains elusive. Here, a metabolomics study using LC-MS and ^1^H NMR spectroscopy was performed to analyze the metabolomic differences in the milk between HS-free and HS dairy cows, and discover diagnostic biomarkers and changes in the metabolic pathway. A total of 53 discriminating metabolites were significantly up- or down-regulated in the HS group compared with the HS-free group (*P* < 0.05). These biomarkers were involved in pathways of carbohydrate, amino acid, lipid, and gut microbiome-derived metabolism. Comparing these potential biomarkers with previously identified HS candidate biomarkers in plasma, significant correlations between the levels of lactate, pyruvate, creatine, acetone, β-hydroxybutyrate, trimethylamine, oleic acid, linoleic acid, lysophosphatidylcholine 16:0, and phosphatidylcholine 42:2 in milk and plasma were found, indicating that the blood-milk barrier became leaky and the levels of these 10 biomarkers in milk can reflect HS-induced metabolomic alterations in blood. These novel findings can support more in-depth research to elucidate the milk-based changes in metabolic pathways in HS lactating dairy cows.

Lactating dairy cows under heat stress (HS) experience limited energy intake, because they cannot meet their bodies’ demands to produce milk and maintain health, resulting in reduced milk yield and quality and leaving the dairy cows susceptible to diseases^1^,^2^. As a result, large economic losses occur in the dairy industry in many countries, such as China^3^,^4^, the US^5^, and Germany^6^. HS-induced metabolomic disturbances are directly responsible for these losses^1^,^7^. This is particularly important given the trends of increased milk productivity using modern molecular genetic technologies accompanied by the temperature increases arising from global climate change^8^.

An accurate determination of when cows enter HS is complicated because the responses to HS affect not only the energy balance but also water, sodium, potassium and chlorine metabolism^9^. Although the temperature and humidity index (THI) remains the most common indicator for HS, using the THI value of 72 as the threshold of HS onset^10^, it allows only judgments based on the air temperature and humidity, is not an accurate measure of the metabolic alterations in dairy cows under HS, and does not account for cow-specific effects (age and breed), and other environment factors. Indeed, the HS-induced physiological changes in dairy cows are multifactorial^7^. Robust metabolite biomarkers are needed to diagnose the threshold of HS onset, monitor its progression, and provide insights into its physiological mechanisms, thereby enabling the implementation of timely interventions to protect dairy cows from diseases, such as ketosis.

Previous research on the performance of dairy cows focused on the HS-induced changes in their respiration rate, heart rate, milk yield, somatic cell counts, and protein, fat, lactose, basal non-esterified fatty acids (FA), insulin, thyroid, noradrenaline, glucose, and plasma urea nitrogen levels in milk^7^,^11^. However, few studies have addressed the global changes in metabolic pathways, and the mechanisms underlying these changes remain unknown.

Metabolomics represents a powerful platform for acquiring the information from hundreds of thousands of low-molecular weight metabolites in plants, animals, and humans, and can be used to provide a global understanding of the pathophysiological alterations stimulated by environmental changes^12^,^13^,^14^,^15^. In dairy cattle, milk can be very conveniently collected, provides information on the changes in the lactation mechanisms of dairy cows, and can be directly analyzed to determine its nutritional quality. Therefore, milk is regarded as the ideal biological sample for monitoring physiological alterations. Compared to blood sampling, milk sampling is non-invasive and occurs on a daily basis, and thus, dairy cows’ metabolic states can be observed in real time. As a result, cows can be monitored and their HS state determined, and prompt management strategies can be implemented to reduce the impact of HS. Hence, the present study used LC-MS and ^1^H NMR spectroscopy to identify metabolic differences in the milk from mid-lactation cows with and without HS to identify HS biomarkers and explore the alterations in the metabolic pathways of lactating dairy cows in different HS states. LC-MS in multiple reaction monitoring (MRM) mode was also used to verify the reliabilities of the discriminating metabolites. Partial Pearson’s correlation analyses of the candidate biomarkers found in milk with those in blood^16^ were performed to track the causes of the disturbed metabolites in milk. The concentrations of cytokines, c-reactive protein, and cytochrome c were detected using enzyme-linked immunosorbent assays (ELISAs) to obtain insights into HS-induced inflammation and apoptosis. Overall, the results of this study improve our understanding of the metabolic alterations in dairy cows exposed to HS. An overview of the study design is shown in Fig. 1.

## Results

### Comparison of the metabolic profiles of HS-free and HS cows

**Figure 2:**
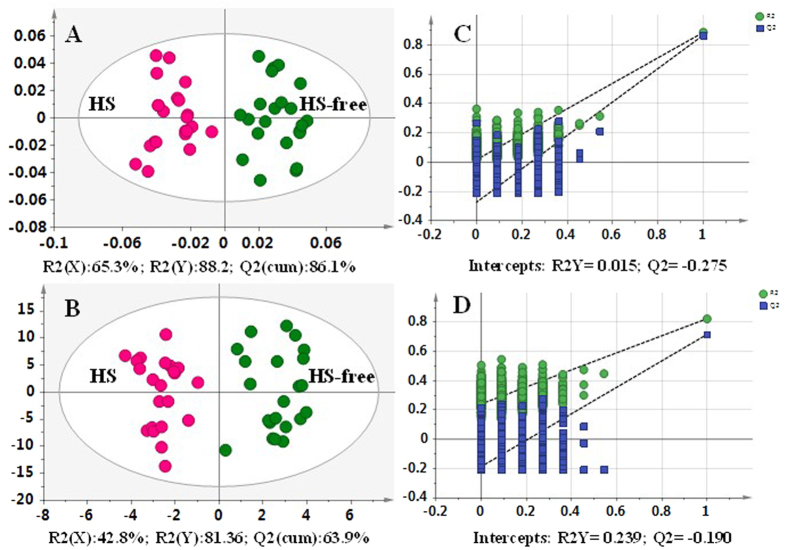
Differentiation of the HS-free and HS groups using multivariate analysis. (**A**) OPLS-DA plots of the ^1^H NMR data of milk serum and fat. (**B**) OPLS-DA plots of the LC-MS data for the milk metabolome. (**C**,**D**) Validation plots of the partial least squares discriminant analysis (PLS-DA) models acquired through 999 permutation tests for the ^1^H NMR data of milk serum and fat and the LC-MS data of milk metabolome, respectively.

The orthogonal partial least squares discriminate analysis (OPLS-DA) plots of the metabolomic data showed a clear separation between the HS-free and HS groups, without any overlap (Fig. 2A,B), indicating that the metabolic profile was significantly altered in the HS milk samples. The ^1^H NMR data of the milk serum and fat (Fig. 2A) and LC-MS data of the milk metabolome (Fig. 2B) for the HS-free and HS groups identified one predictive component and two orthogonal components with satisfactory modelling and predictive abilities of 81.36% < *R*^2^ (Y) <88.2% and 63.9% < *Q*^2^ (cum) <86.1%. To avoid model overfitting, cross-validation across three components with 999 random permutation tests was performed and produced intercepts of 0.015 < *R*^2^ < 0.239 and −0.275 < *Q*^2^ < −0.190 for all of the data (Fig. 2C,D), indicating that the model was valid^16^,^17^,^18^.

### Identification of metabolic candidates and their structures

**Figure 3:**
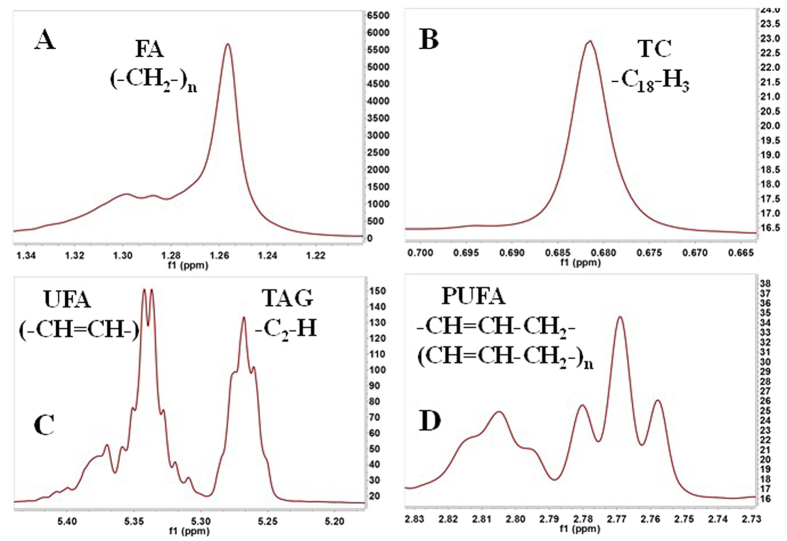
Sections of the ^1^H NMR spectra of a milk fat extract from a representative sample. (**A**) FA, fatty acids. (**B**) TC, total cholesterol. (**C**) UFA, unsaturated fatty acids; TAG, triacylglycerides. (**D**) PUFA, polyunsaturated fatty acids.

By analyzing the ^1^H NMR data, we identified a total of 25 metabolic candidates, which are displayed in Table 1. These metabolites were carbohydrate, amino acid, lipid, and gut microbiome-derived metabolites, suggesting that these metabolic pathways were altered in the HS group. Figure 3 shows an example of the ^1^H NMR spectra of the metabolites identified from milk fat.

Analyzing the LC-MS data revealed that 119 metabolites differed between the HS-free and HS groups, with 33 of these metabolites remaining after the elimination of redundant variables using a combination of extracted ion chromatograms and the R-package Collection of Algorithms for Metabolite Profile Annotation (CAMERA)^16^,^19^. To verify these 33 metabolites, a confirmatory detection was performed using LC-MS in MRM mode. Ultimately, 28 candidates were verified and identified (Table 2). With the exception of urea, the other 27 compounds were lipid metabolites, indicating that lipid metabolism was disturbed in the HS group.

### Metabolic alterations

**Figure 4:**
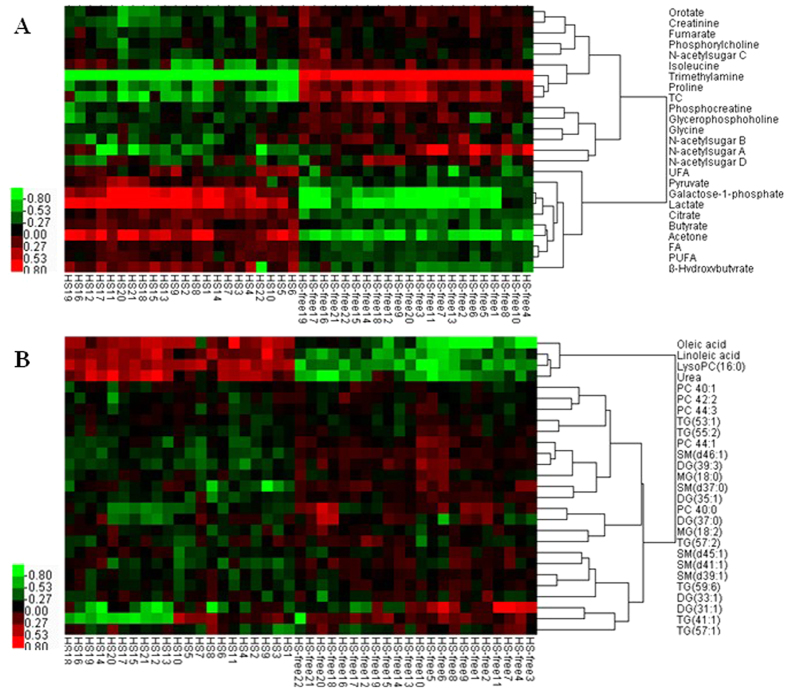
Heat map representation of the unsupervised hierarchical clustering of the (**A**) ^1^H NMR data and (**B**) LC-MS data from the HS and HS-free samples. The shades of red and green represent increases and decreases in the metabolite concentration, respectively, relative its level in the HS-free group. Rows: metabolites; columns: samples.

The ^1^H NMR-identified metabolites that differed between the HS and HS-free groups are shown in Table 1, and a heatmap was constructed based on the alterations in these potential candidates (Fig. 4A). HS-induced alterations in carbohydrate-related metabolites were observed for lactate, pyruvate, galactose-1-phosphate, and citrate, the concentrations of which were increased by 1.27- to 1.79-fold in the HS group compared with the HS-free group (*P* < 0.002), with the exception of fumarate decreased by 0.78 fold in the HS compared with the HS-free group (*P* < 0.001). The concentrations of isoleucine, proline, orotate, glycine, and phosphocreatine, which are metabolites related to protein or amino acids, were changed by 0.69- to 0.88-fold in the HS group compared with the HS-free group (*P* < 0.001). Creatine was increased by 1.59-fold in the HS group compared with the HS-free group (*P* < 0.001). The concentrations of butyrate, acetone, β-hydroxybutyrate (BHBA), FA, PUFA, and UFA, lipid-related metabolites, were increased by 1.08- to 1.38-fold in the HS group compared with the HS-free group (*P* < 0.02). The concentrations of N-acetylsugar A, N-acetylsugar B, N-acetylsugar C, N-acetylsugar D, and TC, were changed by 0.68- to 0.91-fold in the HS group compared with the HS-free group (*P* < 0.01). HS-induced perturbations of the gut microbiome-derived metabolism were also identified, with the concentrations of trimethylamine (TMA) changed by 0.52-fold in the HS group compared with the HS-free group (*P* < 0.001).

The LC-MS-identified metabolites changed by HS are shown in Table 2, and a heatmap was constructed based on the alterations in these potential candidates between the HS-free and HS groups (Fig. 4B). Most of these metabolites are lipids, with the concentrations of oleic acid, linoleic acid, and lysophosphatidylcholine (lysoPC) 16:0 being changed 1.28- to 1.41-fold in the HS group compared with the HS-free group (*P* < 0.005), and phosphatidylcholine (PC), sphingomyelin (SM), monoacylglycerol (MG), diacylglycerol (DG), and triradylglycerol (TG) changed 0.48- to 0.83-fold in the HS group compared with the HS-free group (*P* < 0.02). The concentration of urea, which is related to amino acids, was increased by 1.32-fold in the HS group compared with the HS-free group (*P* < 0.01).

### Partial Pearson’s correlations between the candidate plasma and milk metabolites with corrections for the treatment groups (HS and HS-free)

Table 3 lists the correlation coefficients of candidate metabolites in plasma and milk with corrections for the treatment groups (HS and HS-free). Significant correlations were observed between the lactate, pyruvate, creatine, acetone, BHBA, and TMA levels in plasma and in milk (*P* < 0.001). The significance of the correlations for oleic acid, linoleic acid, lysoPC 16:0, and PC 42:2 was less than 0.01. Moreover, significant correlations were also detected between the lactate and pyruvate levels, and acetone and BHBA levels in milk and plasma (*P* < 0.01).

### Alterations in the cytokine, c-reactive protein, and cytochrome c levels between the HS and HS-free groups

Supplementary Table S6 lists changes in the concentrations of TNF-α, IL-2, IL-10, IL-12, IL-15, c-reactive protein, p53, Bax, Bcl-2, cytochrome c, caspase-3, caspase-8, and caspase-9 in plasma and milk from the HS and HS-free groups. The levels of all of these proteins were significantly higher in the HS group than in the HS-free group (*P* < 0.05), with the exception of Bcl-2 and IL-12 being significantly lower in the HS group than in the HS-free group (*P* < 0.05).

### Partial Pearson’s correlations between the rectal temperature and cytokine, c-reactive protein, cytochrome c, and candidate metabolite levels with corrections for the treatment groups (HS and HS-free)

Supplementary Table S7-S8 list the correlation coefficients between the rectal temperature and candidate metabolite levels in plasma and milk, respectively. Significant correlations were observed between the lactate, pyruvate, creatine, proline, lysine, glycine, threonine, isoleucine, leucine, ornithine, citrulline, and arginine levels in plasma and the rectal temperature (*P* < 0.01), as well as between the lactate, pyruvate, creatine, and citrate levels in milk and the rectal temperature (*P* < 0.01). Supplementary Table S9 displays the correlation coefficients for the cytokine and candidate metabolite levels in plasma and milk. Significant correlations were noted between p53, Bax, Bcl-2, and cytochrome c and acetone and BHBA in both plasma and milk (*P* < 0.01). Supplementary Table S10 lists the significant correlations between the cytochrome c and c-reactive protein levels in plasma and milk (*P* < 0.01).

## Discussion

In the present study, 53 potential metabolite biomarkers that could be used to diagnose the HS states of lactating dairy cows were identified. These potential biomarkers were involved in pathways for carbohydrate, amino acid, lipid, and gut microbiome-derived metabolism, indicating that these metabolic pathways were influenced by HS.

HS up-regulated the pyruvate and lactate levels in the milk from HS dairy cows in the present metabolomics study, as observed in our previous metabolomics study of the plasma from HS dairy cows^16^. Correlation analyses (Table 3) of the pyruvate or lactate levels in plasma and milk from HS and HS-free cows revealed that the plasma pyruvate and lactate concentrations were significantly correlated with their respective concentrations in milk, indicating that these two metabolites are directly secreted from the blood into the milk via the mammary gland^20^.

The milk citrate concentration was increased in the HS group compared with the HS-free group. This species has been used as a biomarker of energy balance in dairy cows and is correlated with the presence of ketone bodies in milk and *de novo* FA synthesis^21^. Because the mammary epithelium is impermeable to citrate in both directions, the milk citrate levels reflect an HS-induced disturbance in mammary function rather than a disturbance in general metabolism. The insignificant differences in the plasma citrate levels between the HS and HS-free groups noted in our previous study^16^ further confirm the hypothesis that the milk citrate levels reflect an HS-induced disturbance in mammary function rather than alterations in the blood citrate metabolism. The reduced fumarate concentration in the HS milk samples may have been caused by a disturbed energy metabolism and impaired function of the tricarboxylic acid cycle^22^.

Lactose is the predominant sugar in most species’ milk. The role of glucose metabolism in milk production is to transport glucose from the blood into the mammary gland for lactose synthesis, and has a vital function in milk yield relating to the osmoregulation of milk^23^,^24^. Although the present study did not identify significant differences in the milk lactose concentrations between the HS and HS-free groups, the milk yields of the HS group were greatly reduced (Supplementary Table S4) relative to the HS-free group (*P* < 0.01). As a result, lactose yields were reduced, suggesting that less blood glucose was available to the mammary glands or lactose synthesis was disrupted.

The increased galactose-1-phosphate concentration in milk was likely attributable to leakage of this component from the mammary epithelial cells into milk, possibly through cell apoptosis, and seems to be strongly dependent on the energy balance states^25^. A negative energy balance (NEB) can greatly increase the apoptotic index in the mammary gland^25^. HS dairy cows are in a NEB state, which may have caused the increased galactose-1-phosphate levels in milk in the present study. The decreased N-acetylsugar A, N-acetylsugar B, N-acetylsugar C, and N-acetyl sugar D levels in the HS group indicated that HS altered their synthesis by reducing the levels of their precursors or influencing the related enzymes.

Decreased concentrations of isoleucine, proline, orotate, glycine, and phosphocreatine were detected in the milk samples from the HS group compared with those from the HS-free group. However, the concentrations of these metabolites were up-regulated in the plasma of the HS dairy cows in our previous metabolomics study^16^. These amino acids may be the main precursors for glucose production via gluconeogenesis or energy production through deamination and oxidation^26^, possibly decreasing their distribution into milk via the mammary gland. Inside the cell, amino acids are involved in metabolic reactions yielding, *inter alia*, CO_2_, urea, polyamines, and nonessential amino acids (NEAAs). The increased urea concentration in the plasma samples from HS dairy cows indicates that more amino acids were metabolized into urea than into milk proteins inside the mammary gland cells. The rectal temperature was significantly correlated with the proline, lysine, creatinine, threonine, isoleucine, leucine, ornithine, citrulline, arginine, and creatine levels in plasma (Supplementary Table S7), suggesting that HS increased the catabolism of the skeletal muscle protein into amino acids in plasma^27^. Significant correlations were observed between the rectal temperature and lactate and pyruvate levels in both plasma and milk (Supplementary Table S8), indicating enhanced anaerobic glycolysis.

In lactating cows, NEB is associated with the mobilization of the body’s energy reserves, leading to extended lipolysis of adipose tissue and partitioning of non-esterified FA into the bloodstream^25^. This partitioning will influence the FA profile in milk serum, such as by increasing the unsaturated FA (UFA) concentrations, mainly C18:1 (oleic acid) and C18:2 (linoleic acid). The same trend was also observed in our results for the milk fat composition, which showed a higher concentration of UFA and polyunsaturated FA (PUFA) in the milk of the cows in the HS group. In cows, the presence of UFA has been related to inflammatory diseases, such as mastitis and metritis. Cholesterol is transported throughout the body via lipoprotein particles. A previous study on the plasma of lactating dairy cows under HS showed that HS reduced the concentrations of high-density lipoprotein (HDL) and very-low-density lipoprotein/low-density lipoprotein (VLDL/LDL), decreasing their supply to the mammary cells^16^. The concentration of cholesterol in milk fat was lower in the HS group than in the HS-free group, indicating that the synthesis and transport of cholesterol into milk is limited under HS.

The BHBA and acetone concentrations were significantly increased in the HS group compared with the HS-free group. These changes agreed with our previous plasma metabolomics study of HS dairy cows^16^. The partial correlation analysis revealed that the BHBA and acetone concentrations in the milk of lactating dairy cows in the HS and HS-free states were significantly correlated with their plasma concentrations reported in a previous study (*P* < 0.01)^16^, indicating that the BHBA and acetone in the blood passed unchanged into the milk via the mammary cells. Thus, elevated concentrations of BHBA and acetone in milk can indicate increased blood BHBA and acetone levels, which are well-known markers of the energy status in dairy cows and reflect excessive protein mobilization and insufficient glucose supply^21^.

Increased P53 protein, Bax, cytochrome c, caspase-3, caspase-8, caspase-9, and decreased Bcl-2, all of which are apoptosis-related molecules^28^, were detected in both plasma and milk of HS group. Increased P53 protein can facilitate the biosynthesis of routing-proteins for apoptosis. The balance of Bcl-2 and Bax ratio acts as an important regulator of the mitochondrial response to apoptotic signals, and its imbalance can lead to cytochrome c release, predicting cell apoptosis. The shear activation of caspase-8 can initiate caspase cascade reaction. Caspase-3 and caspase-9 are regarded as apoptosis executioner and initiator, respectively. The BHBA and acetone concentrations were significantly correlated with P53 protein, Bax, Bcl-2 and cytochrome c in both plasma and milk (Supplementary Table S9), suggesting that the NEB increased apoptosis^29^.

The concentrations of PC, including PC 40:1, PC 40:0, PC 42:2, PC 44:3, and PC 44:1, were lower in the HS group than in the HS-free group, whereas the concentrations of lysoPC 16:0 were higher in the HS group than in the HS-free group. LysoPCs are metabolites of PC catabolism, which is regulated by phospholipases A_1_, A_2_, and D^30^,^31^. The observed alterations in the PC and lysoPC concentrations were consistent with our previously plasma metabolomics study of HS dairy cows^16^. Other lipid-related metabolites in milk, such as SM, MG, DG, and TG were also down-regulated in the HS group. Taken together, these findings clearly show that HS induces overall alterations in the lipid metabolism in both the blood and milk of lactating dairy cows.

There was a strong correlation between the TMA levels in milk and plasma, indicating that alterations in the plasma TMA levels^16^ influenced its concentration in milk (Table 3). We found that HS caused a marked reduction in the milk TMA levels in HS cows compared with the HS-free cows (Table 1). This leads us to believe that HS induced variations in the numbers and/or activities of intestinal microbes.

The immune function of cows can be affected by HS^32^. Increased c-reactive protein, TNF-α, IL-2, IL-10, IL-15, and decreased IL-12 in both the plasma and milk of HS group are regarded as a response to HS-induced inflammation^29^,^33^,^34^, confirming the impairment of the immune system in the HS group. The plasma levels of cytochrome c and c-reactive protein were significantly correlated with their respective concentrations in milk (Supplementary Table S10), suggesting that HS induced an inflammatory response of the whole body of dairy cows^33^. During a mammary immune response, the blood-milk barrier is compromised^35^. Therefore, our previously reported up- or down-regulation of the lactate, pyruvate, creatine, acetone, BHBA, TMA, oleic acid, linoleic acid, lysoPC 16:0, and PC 42:2 levels in the plasma^16^ were also observed in milk in the present study (Table 3). Although the levels of these 10 candidate biomarkers in milk were significantly correlated with their respective concentrations in blood, the remaining 43 potential biomarkers (Tables 1 and 2) in milk did not show similar correlations, indicating that the former 10 biomarkers can reflect HS-induced perturbations in blood metabolites, whereas the remaining 43 reflect a disturbed mammary function rather than general metabolic disturbances.

## Conclusion

Because the levels of the candidate biomarkers lactate, pyruvate, creatine, acetone, BHBA, TMA, oleic acid, linoleic acid, lysoPC 16:0, and PC 42:2 in milk showed strong correlations with their respective concentrations in the blood of HS-free and HS dairy cows, the detection of these milk metabolites can diagnose HS states and HS-induced perturbations in general metabolism. Other potential biomarkers can reflect HS-induced alterations in the mammary function of HS dairy cows. The results identify potential biomarkers that could be used to accurately monitor the HS states of lactating dairy cows and merit further investigation to elucidate the physiological mechanisms underlying the HS-induced changes in metabolic pathways. This could lead to better management practices for dairy cows exposed to HS.

## Materials and Methods

All experiments involving animals were conducted according to the principles of the Chinese Academy of Agricultural Sciences Animal Care and Use Committee (Beijing, China), which approved the study protocols.

### Chemicals and reagents

Deuterium oxide and deuterated chloroform were purchased from USA Cambridge Isotope Laboratories, Inc. (Tewksbury, MA, USA); and 3-(trimethylsilyl) propionic-2,2,3,3,d4 propionic acid sodium salt was purchased from Merck Canada Inc. (Kirkland, QC, Canada). HPLC-grade methanol, methyl tert-butyl ether, water, formic acid, and ammonium formate were purchased from Merck (Darmstadt, Germany).

### Sample collection

According to NRC (1971), THI was calculated using the formula: THI = (1.8 × T_db_ + 32) −[(0.55−0.0055 × RH) × (1.8 × T_db_ −26.8)], where T_db_ is the dry-bulb temperature (°C) and RH is the relative humidity (%). Forty-four Holstein cows in second parity at mid-lactation that were fed the same diet were used in this trial. The HS-free group consisted of 22 cows, with milk samples obtained in the spring season under a THI of 50–55 for one month. The HS group consisted of 22 cows, with samples obtained in the summer season, after THI gradually increased from 68 to 80 over 1 month and remained stable at 80 for 1 week. Morning milk samples were collected before feeding, and stored at −80 °C until further use. The detailed characteristics of the selected dairy cows, feed composition, temperature, humidity, rectal temperature, respiration rate, and production characteristics of the two groups of dairy cows are shown in Supplementary Table S1–S4 referenced to the reported paper^16^.

### NMR spectroscopic analysis of milk serum and fat

Milk samples were thawed at room temperature, homogenized, and centrifuged at 20000 g for 30 min at 4 °C. All the samples were analyzed at 298 K using a VARIAN VNMRS 600 MHz NMR SPECTROMETER (Varian Inc., Palo Alto, CA) operating at 599.871 MHz using a 5-mm inverse-proton (HX) triple resonance probe with z-axis gradient coil. The ^1^H NMR spectra of the milk serum were recorded using the water-suppressed standard 1D CPMG pulse sequence (RD-90°-(τ-180°-τ)n-ACQ), where a fixed total spin–spin relaxation delay 2nτ of 320 ms was applied to attenuate the broad NMR signals from slowly tumbling molecules (such as proteins) and retain those from low-molecular weight compounds and some lipid components^36^. The free induction decays (FIDs) were collected via 64 K data points with a spectral width of 12000 Hz and 128 scans. The FIDs were zero-filled to double size and multiplied by an exponential line-broadening factor of 0.5 Hz before Fourier transformation (FT). The ^1^H NMR spectra of milk fat do not require presaturation of the water resonance. Here, we used a simple 901 pulse-acquire sequence because we were measuring fully relaxed spectra. We collected 64 transients into 32,768 data points; the other parameters were as follows: relaxation delay = 2 s, spectral width = 20 ppm, and acquisition time = 1.36 s. The metabolites were identified by inserting the experimental spectra into the Chenomx spectral database (Edmonton, AB, Canada), and comparing them with the spectra of standard compounds.

### LC-MS analysis of milk serum

Fifty microliters (50 μL) of milk was mixed with 350 μL of solvents composed of methanol and methyl tert-butyl ether (MTBE) in a 1:1 (v:v) ratio, and vitamin E acetate was added as an internal standard (IS), at a final concentration of 25 ppm. The solution was filtered through a Captiva 96-well filter plate (Agilent, Santa Clara, CA, USA). LC-MS/MS analyses were performed using Shimadzu ultra-fast LC (UFLC) 20ADXR system and a 5600 Triple TOF mass spectrometer (Applied Biosystems/MDS Sciex, Concord, ON, Canada). Five microliters (5 μL) of prepared samples was injected into a column at 40 °C (ACQUITY UPLC HSS T_3_ 1.8 μm, 2.1 × 100 mm column, Waters, Dublin, Ireland). The temperature of auto-sampler was maintained at 15 °C. The gradient consisted of mobile phase A (10 mM ammonium formate in water) and mobile phase B (10 mM ammonium formate in methanol) pumped at 0.3 mL/min within a total run time of 33 min. The linear gradients were: 70% B at 1 min, 99% B at 15 min, 99% B at 20 min, 1% B at 20.1 min, and 1% B at 33 min. The electrospray ionization (ESI) source was set up in positive ionization mode. The MS parameters for detection were as follows: ESI source voltage 5.5 kV; vaporizer temperature, 550 °C; drying gas (N_2_) pressure, 60 psi; nebulizer gas (N_2_) pressure, 60 psi; curtain gas (N_2_) pressure, 30 psi; and declustering potential, 50 V. The scan range was *m/z* 60–1,000. Data acquisition and processing were performed using Analyst® TF 1.6 Software (Applied Biosystems/MDS Sciex). Auto-calibration was performed using the Calibrant Delivery System in both positive and negative ion modes. The metabolites were identified using the information-dependent acquisition mode in the MS/MS analyses. The collision energy was 35 eV. High-resolution MS, isotope abundance ratios, MS/MS, the Human Metabolome database, the METLIN database^37^, a literature search, and comparisons with standards were employed to identify the ion structures. Quality control (QC) samples were detected once every 10 LC-MS runs to monitor the reproducibility of the instrument. The plasma samples from the different groups were randomly alternated during the analysis. Auto-calibration was performed using the Calibrant Delivery System in both positive and negative ion modes.

### Multivariate data analysis

The raw ^1^H NMR spectra were manually corrected for phase and baseline distortions using TopSpin software (version 3.0; BrukerBiospin) and were referenced to the anomeric doublet resonance of lactose (δ 5.233 ppm) for the milk serum spectra and the 3-(trimethylsilyl) propionic-2,2,3,3,d4 acid sodium salt (δ 0.0 ppm) signal for the milk fat spectra. The ^1^H NMR spectra of all specimens were binned into 0.001 ppm integral regions and integrated over the 0.5–7.0 ppm region with the exclusion of water (δ 4.7–5.12) using the AMIX software package (version 3.8.3, BrukerBiospin). The spectra were normalized to the total sum of the spectral integrals to compensate for differences in sample concentrations. The two-dimensional data matrices from the milk serum and fat data were integrated into one Excel file for the subsequent SIMCA-P analysis.

The raw LC-MS data files (.wiff) were converted into mzXML format using ProteoWizard (http://metlin.scripps.edu/xcms/download/pwiz/pwiz.zip). The files were processed using an open-source XCMS package (version 1.20.1) in R statistical software (version 2.10.0) for peak discrimination, filtering and alignment^38^. During XCMS implementation, the R-package CAMERA was used to annotate the isotope, adduct, and product ion peaks. The resulting two-dimensional matrices, including the observations (sample names) in columns, the variables (*m/z*-retention time pairs) in rows, and the peak areas, were saved as CSV files. The IS was used for data quality control (reproducibility) and normalization.

The resulting data sets were then imported into the SIMCA-P 13.0 software package (Umetrics AB, Umeå, Sweden); pareto and centre scaling were applied to the LC-MS and ^1^H NMR data, respectively, to reduce the noise and artifacts in the models. Principal component analysis (PCA) was performed on the scaled data to visualize the global clustering and separation trends or outliers. PLS-DA models were applied to validate the model against overfitting through 999 random permutation tests. Discriminating variables were selected according to the S-plots, variable importance in projection values (VIP >1), and raw data plots in the OPLS-DA models^16^,^17^,^18^. Furthermore, independent *t*-tests (*P* < 0.05) (SPSS version 13.0) were used to determine the significance of each metabolite in discriminating the HS group from the HS-free groups.

To analyze the correlations between the levels of all the potential biomarkers discovered in milk with their respective concentrations in plasma reported previously from specimens collected at the same time, partial Pearson’s correlation coefficients were calculated at two confidence levels (0.01 and 0.001) by correcting for the HS group in the first order using the ParCorA software program, which can be downloaded at http://mendes.vbi.vt.edu/tiki-index.php?page. Cluster 3.0 and Treeview were separately used for cluster analysis and visualization, respectively.

### UFLC-MS/MS-based verification test

The chromatographic gradient elution conditions were unchanged. The eluent was injected into a triple quadrupole-trap mass spectrometer equipped with an ESI source (QTRAP 5500, Applied Biosystems/MDS Sciex). All of the potential candidates discovered by LC-MS were detected by UFLC-MS/MS in MRM mode. For each analyte of interest, the collision energies and precursor/fragment ion pairs were pre-optimized to generate an optimal signal-to-noise ratio. The MRM transitions are listed in Supplementary Table S1.

### ELISAs for cytokines, c-reactive protein, and cytochrome c

The cytokines, c-reactive protein, and cytochrome c concentrations in both plasma and milk were analyzed using commercially available bovine-specific ELISA kits (Abcam^®^, Cambridge, MA, USA), according to the manufacturers’ instructions.

## Additional Information

**How to cite this article**: Tian, H. *et al*. Integrated Metabolomics Study of the Milk of Heat-stressed Lactating Dairy Cows. *Sci. Rep*. **6**, 24208; doi: 10.1038/srep24208 (2016).

## Supplementary Material

Supplementary Information

## Figures and Tables

**Figure 1:**
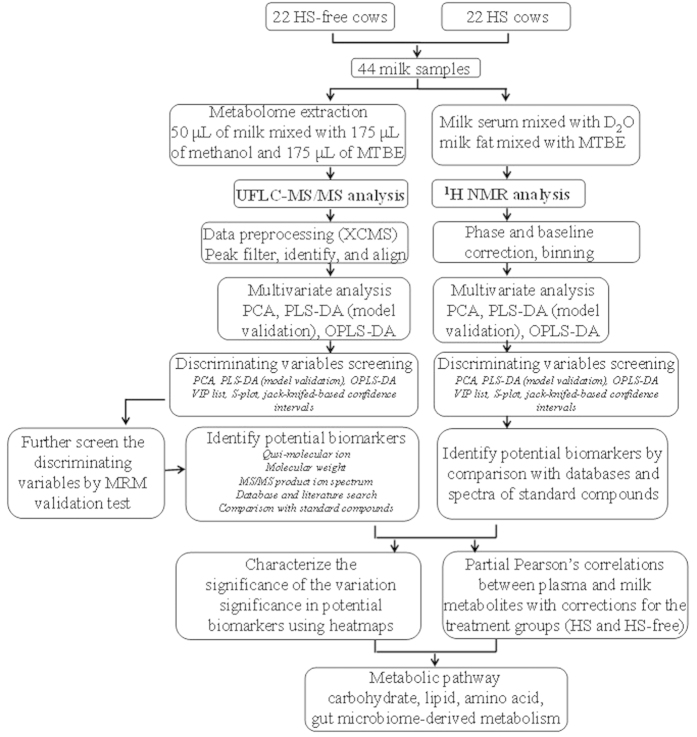
Overview of the strategy used to identify diagnostic biomarkers of HS in lactating dairy cows. HS, heat stress; MTBE, methyl tert-butyl ether; NMR, nuclear magnetic resonance; MVDA, multivariate statistical data analysis; PCA, principal component analysis; PLS-DA, partial least squares discriminant analysis; OPLS-DA, orthogonal partial least squares discriminate analysis.

**Table 1:** Diagnostic biomarkers identified by ^1^H NMR and comparison of their levels in the HS and HS-free groups.

Metabolic pathway	Metabolite	Chemical shift (ppm)	Assignment^a^	*P*-value^b^	FC^c^
Carbohydrate	Lactate	1.33	CH_3_ (d)	<0.001	1.46
Carbohydrate	Pyruvate	2.41	CH_3_ (s)	<0.002	1.32
Carbohydrate	Citrate	2.74	CH_2_ (d)	<0.001	1.79
Carbohydrate	Galactose-1-phosphate	5.51	CH (dd)	<0.001	1.27
Carbohydrate	Fumarate	6.53	2 × CH (s)	<0.001	0.78
Amino acid	Isoleucine	1.00	CH_3_ (d)	<0.001	0.88
Amino acid	Creatine	3.03	CH_3_ (s)	<0.001	1.59
Amino acid	Phosphocreatine	3.05	CH_3_ (s)	<0.001	0.69
Amino acid	Glycine	3.57	CH_2_ (s)	<0.001	0.83
Amino acid	Proline	4.13	CH (m)	<0.001	0.81
Amino acid	Orotate	6.20	CH (s)	<0.001	0.72
Lipid	TC	0.68	18 × CH_3_ (s)	<0.001	0.91
Lipid	Butyrate	0.91	CH_3_ (t)	<0.001	1.23
Lipid	FA	1.26	n × CH_2_ (s)	<0.001	1.14
Lipid	N-acetylsugar A	2.04	CH_3_ (s)	<0.001	0.86
Lipid	N-acetylsugar B	2.06	CH_3_ (s)	<0.001	0.71
Lipid	N-acetylsugar C	2.07	CH_3_ (s)	<0.001	0.68
Lipid	Acetone	2.24	2 × CH_3_ (s)	<0.001	1.42
Lipid	β-Hydroxybutyrate	2.36	CH_2_ (m)	<0.001	1.38
Lipid	PUFA	2.79	n × CH (t)	<0.001	1.08
Lipid	Phosphorylcholine	3.22	3 × CH_3_ (s)	<0.001	0.84
Lipid	Glycerophosphoholine	3.23	3 × CH_3_ (s)	<0.001	0.89
Lipid	UFA	5.34	n × CH (m)	<0.02	1.18
Lipid	N-acetylsugar D	5.41	CH (m)	<0.01	0.85
Gut microbiome-derived metabolism	Trimethylamine	2.85	3 × CH_3_ (s)	<0.001	0.52

^a^d, doublet; s, singlet; dd, double doublet, m, multiplet; t, triplet; q, quartet.

^b^*P*-value, independent *t*-test for HS-free versus HS.

^c^FC, fold change in the metabolite concentration (HS/HS-free).

**Table 2:** Diagnostic biomarkers identified by LC-MS/MS and comparison of their levels in the between HS and HS-free groups.

Metabolic pathway	Metabolite	Adduct	Identification *m/z*, Retention Time	*P*-value^a^	FC^b^
Amino acid	Urea	2 M + H	121.0725, 1.18	<0.01	1.32
Lipid	Linoleic acid	M + Na	303.2295, 4.96	<0.001	1.35
Lipid	Oleic acid	M + Na	305.2452, 5.13	<0.001	1.41
Lipid	LysoPC 16:0	M + H	496.3399, 7.93	<0.005	1.28
Lipid	PC 40:1^c^	M + H	732.5544, 18.42	<0.02	0.48
Lipid	PC 42:2^c^	M + H	758.5690, 18.93	<0.01	0.82
Lipid	PC 44:3^c^	M + H	784.5842, 19.08	<0.01	0.79
Lipid	PC 40:0^c^	M + H	734.5712, 19.57	<0.01	0.57
Lipid	PC 44:1^c^	M + H	788.6172, 21.23	<0.02	0.68
Lipid	SM d37:0^d^	M + H	677.5587, 17.28	<0.002	0.83
Lipid	SM d39:1^d^	M + H	703.5738, 18.08	<0.01	0.77
Lipid	SM d41:1^d^	M + H	731.6054, 19.53	<0.005	0.69
Lipid	SM d45:1^d^	M + H	787.6699, 22.38	<0.01	0.59
Lipid	SM d46:1^d^	M + H	801.6844, 23.19	<0.002	0.61
Lipid	MG 18:2^e^	M + H	355.2837, 21.80	<0.01	0.80
Lipid	MG 18:0^e^	M + Na	381.2978, 23.02	<0.01	0.54
Lipid	DG 31:1^f^	M + NH_4_	528.4626, 17.47	<0.01	0.73
Lipid	DG 35:1^f^	M + NH_4_	584.5245, 20.31	<0.02	0.72
Lipid	DG 37:2^f^	M + Na	615.4955, 20.79	<0.01	0.85
Lipid	DG 39:3^f^	M + NH_4_	636.5572, 21.05	<0.01	0.64
Lipid	DG 33:1^f^	M + H-H_2_O	521.4563, 21.34	<0.02	0.68
Lipid	DG 37:0^f^	M + H-H_2_O	579.5343, 23.07	<0.01	0.77
Lipid	TG 57:2^g^	M + NH_4_	904.8320, 22.03	<0.01	0.66
Lipid	TG 57:1^g^	M + NH_4_	906.8482, 22.80	<0.01	0.79
Lipid	TG 53:1^g^	M + NH_4_	850.7848, 23.03	<0.005	0.58
Lipid	TG 55:2^g^	M + NH_4_	876.8019, 24.03	<0.01	0.52
Lipid	TG 41:1^g^	M + NH_4_	708.6133, 24.52	<0.01	0.81
Lipid	TG 59:6^g^	M + H	907.7736, 25.20	<0.01	0.75

^a^*P*-value of independent *t*-test for HS-free versus HS.

^b^Fold change in the metabolite concentration (HS/HS-free).

^c^Phosphatidylcholine.

^d^Sphingomyelin.

^e^Monoacylglycerol.

^f^Diacylglycerol.

^g^Triacylglycerol.

**Table 3:** Partial Pearson’s correlations between the candidate metabolites in plasma and milk with corrections for the treatment groups (HS and HS-free).

	Plasma lactate	Plasma pyruvate	Plasma creatine	Plasma acetone	Plasma BHBA	Plasma TMA	Plasma oleic acid	Plasma linoleic acid	Plasma lysoPC 16:0	Plasma PC 42:2
Milk lactate	0.88**	0.76*	NS	NS	NS	NS	NS	NS	NS	NS
Milk pyruvate	0.63*	0.82**	NS	NS	NS	NS	NS	NS	NS	NS
Milk creatine	NS	NS	0.76**	NS	NS	NS	NS	NS	NS	NS
Milk acetone	NS	NS	NS	0.89**	0.79*	NS	NS	NS	NS	NS
Milk BHBA	NS	NS	NS	0.85*	0.83**	NS	NS	NS	NS	NS
Milk TMA	NS	NS	NS	NS	NS	0.92**	NS	NS	NS	NS
Milk oleic acid	NS	NS	NS	NS	NS	NS	0.78*	NS	NS	NS
Milk linoleic acid	NS	NS	NS	NS	NS	NS	NS	0.81*	NS	NS
Milk lysoPC (16:0)	NS	NS	NS	NS	NS	NS	NS	NS	0.84*	NS
Milk PC 42:2	NS	NS	NS	NS	NS	NS	NS	NS	NS	0.92*

*P < 0.01. **P < 0.001. NS, no significant correlations.

## References

[b1] Bernabucci U. . The effects of heat stress in Italian Holstein dairy cattle. J. Dairy Sci. 97, 471–486 (2014). 24210494 10.3168/jds.2013-6611

[b2] West J. W. Effects of heat-stress on production in dairy cattle. J. Dairy. Sci. 86, 2131–2144 (2003). 12836950 10.3168/jds.S0022-0302(03)73803-X

[b3] Wang J. P. . Effect of saturated fatty acid supplementation on production and metabolism indices in heat-stressed mid-lactation dairy cows. J. Dairy Sci. 93, 4121–4127 (2010). 20723687 10.3168/jds.2009-2635

[b4] Cheng J. B. . Natural period change of heat stress reveals unique “heat-stressed milk protein decrease syndrome” in mid-lactation dairy cows. China Animal Husbandry & Veterinary Medicine 41, 73–84 (2014).

[b5] St-Pierre N. R., Cobanov B. & Schnitkey G. Economic losses from heat stress by US livestock industries. J. Dairy Sci. 86, E52–E77 (2003).

[b6] Schüller L. K., Burfeind O. & Heuwieser W. Impact of heat stress on conception rate of dairy cows in the moderate climate considering different temperature-humidity index thresholds, periods relative to breeding, and heat load indices. Theriogenology 81, 1050–1057 (2014). 24612695 10.1016/j.theriogenology.2014.01.029

[b7] Wheelock J. B., Rhoads R. P., Vanbaale M. J., Sanders S. R. & Baumgard L. H. Effects of heat stress on energetic metabolism in lactating Holstein cows. J. Dairy Sci. 93, 644–655 (2010). 20105536 10.3168/jds.2009-2295

[b8] Key N. & Sneeringer S. Potential effects of climate change on the productivity of U.S. dairies. Amer. J. Agr. Econ. 1–21 (2014).24839299

[b9] Sevi A. . Effects of solar radiation and feeding time on behavior, immune response and production of lactating ewes under high ambient temperature. J. Dairy Sci. 84, 629–40 (2001). 11286417 10.3168/jds.S0022-0302(01)74518-3

[b10] Hammami H., Bormann J., M’hamdi N., Montaldo H. H. & Gengler N. Evaluation of heat stress effects on production traits and somatic cell score of Holsteins in a temperate environment. J. Dairy Sci. 96, 1844–1855 (2013). 23313002 10.3168/jds.2012-5947

[b11] Vernon R. G. Effects of diet on lipolysis and its regulation. Proc. Nutr. Soc. 51, 397–408 (1992). 1480634 10.1079/pns19920053

[b12] Sreekumar A. . Metabolomic profiles delineate potential role for sarcosine in prostate cancer progression. Nature 457, 910–914 (2009). 19212411 10.1038/nature07762 PMC2724746

[b13] Nie W. . Advanced mass spectrometry-based multi-omics technologies for exploring the pathogenesis of hepatocellular carcinoma. Mass Spectrom Rev. 2, doi: 10.1002/mas.21439. [Epub ahead of print] (2014). 24890331

[b14] Tian Y. . Integrative metabonomics as potential method for diagnosis of thyroid malignancy. Sci. Rep. 21, 14869 (2015). 26486570 10.1038/srep14869 PMC4613561

[b15] Zhao Y. . A metabolomics study delineating geographical location-associated primary metabolic changes in the leaves of growing tobacco plants by GC-MS and CE-MS. Sci. Rep. 9, 16346 (2015). 26549189 10.1038/srep16346 PMC4637841

[b16] Tian H. . Identification of diagnostic biomarkers and metabolic pathway shifts of heat-stressed lactating dairy cows. J. Proteomics 125, 17–28 (2015). 25913299 10.1016/j.jprot.2015.04.014

[b17] An Z. . Integrated ionization approach for RRLC-MS/MS-based metabonomics: finding potential biomarkers for lung cancer. J. Proteome Res. 8, 4071–4081 (2010). 20560663 10.1021/pr100265g

[b18] Li T. . *In situ* biomarker discovery and label-free molecular histopathological diagnosis of lung cancer by ambient mass spectrometry imaging. Sci. Rep. 5, 14089 (2015). 26404114 10.1038/srep14089 PMC4585892

[b19] Tian H. . Plasma metabolome analysis by integrated ionization rapid-resolution liquid chromatography/tandem mass spectrometry. Rapid Commun. Mass Spectrom. 27, 2071–2080 (2013). 23943328 10.1002/rcm.6666

[b20] Lehmann M., Wellnitz O. & Bruckmaier R. M. Concomitant lipopolysaccharide-induced transfer of blood-derived components including immunoglobulins into milk. J. Dairy Sci. 96, 889–896 (2013). 23219120 10.3168/jds.2012-5410

[b21] Klein M. S. . Correlations between milk and plasma levels of amino and carboxylic acids in dairy cows. J. Proteome Res. 12, 5223–5232 (2013). 23931703 10.1021/pr4006537

[b22] Sundekilde U. K., Poulsen N. A., Larsen L. B. & Bertram H. C. Nuclear magnetic resonance metabonomics reveals strong association between milk metabolites and somatic cell count in bovine milk. J. Dairy Sci. 96, 290–299 (2013). 23182357 10.3168/jds.2012-5819

[b23] Eger M. . Glucose transporter expression differs between bovine monocyte and macrophage subsets and is influenced by milk production. J Dairy Sci. 99, 2276–2287 (2016). 26723114 10.3168/jds.2015-10435

[b24] Osorio J. S., Lohakare J. & Bionaz M. Biosynthesis of milk fat, protein, and lactose: roles of transcriptional and post-transcriptional regulation. Physiol Genomics. 2016 Jan 26:physiolgenomics.00016. doi: 10.1152/physiolgenomics.00016.2015. [Epub ahead of print] (2015). 26812986

[b25] Lu J. . Changes in milk proteome and metabolome associated with dry period length, energy balance, and lactation stage in postparturient dairy cows. J. Proteome Res. 12, 3288–3296 (2013). 23738862 10.1021/pr4001306

[b26] Li L. O. . Compartmentalized acyl-CoA metabolism in skeletal muscle regulates systemic glucose homeostasis. Diabetes 64, 23–25 (2015). 25071025 10.2337/db13-1070 PMC4274800

[b27] Wheelock J. B., Rhoads R. P., Vanbaale M. J., Sanders S. R. & Baumgard L. H. Effects of heat stress on energetic metabolism in lactating Holstein cows. J Dairy Sci. 93, 644–655 (2010). 20105536 10.3168/jds.2009-2295

[b28] Cheng C. H. . High temperature induces apoptosis and oxidative stress in pufferfish (Takifugu obscurus) blood cells. J Therm Biol. 53, 172–179 (2015). 26590470 10.1016/j.jtherbio.2015.08.002

[b29] Morris D. G. . Pleiotropic effects of negative energy balance in the postpartum dairy cow on splenic gene expression: repercussions for innate and adaptive immunity. Physiol Genomics 39, 28–37 (2009). 19567785 10.1152/physiolgenomics.90394.2008 PMC2747343

[b30] Dong J. . Lysophosphatidylcholine profiling of plasma: discrimination of isomers and discovery of lung cancer biomarkers. Metabolomics 6, 478–448 (2010).

[b31] Glunde K. & Serkova N. J. Therapeutic targets and biomarkers identified in cancer choline phospholipid metabolism. Pharmacogenomics 7, 1109–1123 (2006). 17054420 10.2217/14622416.7.7.1109

[b32] Tao S. . Effect of heat stress during the dry period on mammary gland development. J. Dairy Sci. 94, 5976–5986 (2011). 22118086 10.3168/jds.2011-4329

[b33] Kaldur T. . Effects of heat acclimation on changes in oxidative stress and inflammation caused by endurance capacity test in the heat. Oxid Med Cell Longev. 2014, 107137 (2014). 24895525 10.1155/2014/107137 PMC4034648

[b34] Caroprese M., Albenzio M., Marino R., Santillo A. & Sevi A. Dietary glutamine enhances immune responses of dairy cows under high ambient temperature. J Dairy Sci. 96, 3002–3011 (2013). 23498025 10.3168/jds.2012-6306

[b35] Lehmann M., Wellnitz O. & Bruckmaier R. M. Concomitant lipopolysaccharide-induced transfer of blood-derived components including immunoglobulins into milk. J. Dairy Sci. 96, 889–896 (2013). 23219120 10.3168/jds.2012-5410

[b36] Beckonert O. . Metabolic profiling, metabolomic and metabonomic procedures for NMR spectroscopy of urine, plasma, serum and tissue extracts. Nat. Protoc. 2, 2692–2703 (2007). 18007604 10.1038/nprot.2007.376

[b37] Zhu Z. J. . Liquid chromatography quadrupole time-of-flight mass spectrometry characterization of metabolites guided by the METLIN database. Nat. Protoc. 8, 451–460 (2013). 23391889 10.1038/nprot.2013.004 PMC3666335

[b38] Smith C. A., Want E. J., O’Maille G., Abagyan R. & Siuzdak G. XCMS: processing mass spectrometry data for metabolite profiling using nonlinear peak alignment, matching, and identification. Anal. Chem. 78, 779–787 (2006). 16448051 10.1021/ac051437y

