# Supplementary material for: Integrated Metabolomics Study of the Milk of Heat-stressed Lactating Dairy Cows
Source: Sci Rep. 2016 Apr 6;6:24208. doi: 10.1038/srep24208 (PMC4822173; doi:10.1038/srep24208)
Supplement: Supplementary Information [file srep24208-s1.pdf]

Integrated Metabolomics Study of the Milk of Heat-stressed Lactating Dairy Cows

He Tian[1,*], Nan Zheng[1, *], Weiyu Wang[2, *], Jianbo Cheng[3], Songli Li[1], Yangdong Zhang[1], Jiaqi Wang[1]

[1]Institute of Animal Science, Chinese Academy of Agricultural Sciences, Beijing, 100193, P.R. China. [2]The High School affiliated to Renmin University of China, Beijing, 100080, P.R. China. [3]College of Animal Science and Technology, Anhui Agricultural University, Hefei, 230036, P.R. China. [*]These authors contributed equally to this work. Correspondence and requests for materials should be addressed to J.W. (email: wang-jia-qi@263.net).

Supplementary Information

1. Table S1 - Ingredients and chemical composition of diets.

2. Table S2 - Temperature and humidity for the dairy cows used in the study.

3. Table S3 - Rectal temperature and respiration rate of dairy cows.

4. Table S4 - Production parameters of dairy cows.

5. Table S5 -Transition list of metabolome candidate ions verified by LC-MS/MS in MRM mode.

6. Table S6 – Concentration alterations of cytokine, cytochrome C, and c-reactive protein between HS-free and HS groups.

7. Table S7 -Partial Pearson’s correlation between the rectal temperature and candidate plasma metabolites with correction for the treatment groups (HS and HS-free).

8. Table S8 -Partial Pearson’s correlation between rectal temperature and candidate milk metabolites with correction for the treatment groups (HS and HS-free).

9. Table S9 -Partial Pearson’s correlation between cytokine and candidate metabolites with correction of the treatment groups (HS and HS-free).

10. Table S10 -Partial Pearson’s correlation between inflammation markers and candidate metabolites with correction of the treatment groups (HS and HS-free).

Table S1 - Ingredients and chemical composition of diets.

Items	% of DM
Ingredient	
Alfalfa hay	14.2
Guinea grass	2.1
Whole plant corn silage	16.5
Oat grass	5.0
Whole cottonseed	5.1
Beet pulp	6.1
Concentrate supplement	51.0
Chemical analysis, % DM	
CP	16.9
NDF	32.8
NE_L, Mcal/kg of DM	1.7
RDP/CP, %	61.2
NEL/CP	10.1
NFC	35.1
Ca	1.11
P	0.43

Table S2 - Temperature and humidity for the dairy cows used in the study.

	Item	HS-free			HS		
		Temp. (^{o}C)	RH (%)	THI	Temp. (^{o}C)	RH (%)	THI
Day 3	a.m. 6:00	10.5	55.3	52.8	30.3	58.5	80.2
	p.m. 2:00	18.5	23.5	62.6	33.2	58.8	84.3
	p.m. 10:00	12.5	57.5	55.5	30.0	62.7	80.4
Day 2	a.m. 6:00	13.1	64.2	56.2	27.3	68.7	77.3
	p.m. 2:00	17.4	38.5	61.8	31.5	62.2	82.5
	p.m. 10:00	12.1	49.3	55.2	26.7	73.8	77.0
Day 1	a.m. 6:00	9.0	69.5	50.0	30.4	59.0	80.4
	p.m. 2:00	13.0	62.0	56.1	33.3	52.7	83.3
	p.m. 10:00	12.0	66.5	54.6	30.6	63.3	81.5
Day 0	a.m. 6:00	11.5	67.4	53.8	31.5	61.0	82.3
	p.m. 2:00	15.7	52.3	59.9	32.9	57.4	83.6
	p.m. 10:00	9.9	59.8	51.8	31.2	62.3	82.1

Day 3, day2, day 1, and day 0 are respective time points before sample collection of dairy cows. RH, relative humidity; Temp., temperature.

Table S3 - Rectal temperature and respiration rate of dairy cows.

Parameters	HS-free	HS	*P*-value
Rectal temperature (°C)			
06:00	38.13	38.79	< 0.01
14:00	38.25	39.21	< 0.01
22:00	38.48	39.45	< 0.01
Respiration rate (breaths/min)			
06:00	50.24	64.42	< 0.01
14:00	50.17	81.75	< 0.01
22:00	49.85	69.25	< 0.01

Table S4 - Production parameters of dairy cows.

Parameter	HS-free	HS	*P*-value
DMI, kg/d	20.15 ± 3.21	17.31 ± 2.86	< 0.01
Milk yield, kg/d	38.4 ± 4.9	30.9 ±3.7	< 0.01
Milk component			
Fat, %	3.51 ± 0.32	3.30 ± 0.44	0.39
Protein, %	3.45 ± 0.26	2.90 ± 0.35	< 0.01
Lactose, %	4.86 ± 0.11	4.88 ± 0.13	0.92
MUN[1] (mg/100 mL)	11.76 ± 1.97	13.53 ± 1.46	0.01
SCC[2] ($\times 10^3$/mL)	104.78 ± 20.14	465.63 ± 53.21	< 0.01

[1]MUN, milk urea nitrogen. [2]SCC, somatic cell count.

Table S5 -Transition list of metabolome candidate ions verified by LC-MS/MS in MRM mode.

Ionization mode	RT (min)	Precursor ion (Q1)	Product ion (Q3)
$[2M + H]^+$	1.18	121.1	61.1
$[M + Na]^+$	5.13	305.2	261.2
$[M + Na]^+$	4.96	303.2	259.2
$[M + H]^+$	7.93	496.3	184.1
$[M + H]^+$	18.42	732.6	184.1
$[M + H]^+$	19.57	734.6	184.1
$[M + H]^+$	18.93	758.6	184.1
$[M + H]^+$	19.08	784.6	184.1
$[M + H]^+$	21.23	788.6	184.1
$[M + H]^+$	18.08	703.6	184.1
$[M + H]^+$	22.38	787.7	184.1
$[M + H]^+$	17.28	677.6	184.1
$[M + H]^+$	23.19	801.7	184.1
$[M + H]^+$	19.53	731.6	184.1
$[M + H]^+$	21.80	355.3	109.1
$[M + Na]^+$	23.02	381.3	81.0
$[M + NH_4]^+$	17.47	528.5	339.3
$[M + H - H_2O]^+$	21.34	521.5	57.1
$[M + NH_4]^+$	20.31	584.5	285.2
$[M + Na]^+$	20.79	615.5	359.3
$[M + H - H_2O]^+$	23.07	579.5	239.2
$[M + NH_4]^+$	21.05	636.6	337.3
$[M + NH_4]^+$	24.52	708.6	575.5
$[M + NH_4]^+$	23.03	850.8	577.5
$[M + NH_4]^+$	24.03	876.8	577.5
$[M + NH_4]^+$	22.80	906.8	605.6
$[M + NH_4]^+$	22.03	904.8	605.5
$[M + H]^+$	25.20	907.8	625.5
$[M + H]^+$	16.81	516.4	243.2
$[M + H]^+$	18.37	544.4	243.2
$[M + H]^+$	20.12	558.5	285.2
$[M + H]^+$	19.41	572.5	299.2
$[M + H]^+$	24.19	656.6	383.3

Table S6 – Concentration alterations of cytokine, cytochrome C, and c-reactive protein between HS-free and HS groups.

Proteins (pg/mL)	HS-free	HS	*P*-value
Plasma TNF-α (pg/mL)	176.15 ± 10.21	191.62 ± 15.76	< 0.01
Plasma IL-2 (pg/mL)	391.62 ± 20.13	426.15 ± 26.37	< 0.01
Plasma IL-10 (pg/mL)	51.48 ± 10.96	67.26 ± 8.32	< 0.02
Plasma IL-12 (pg/mL)	95.74 ± 6.73	80.16 ± 8.34	< 0.02
Plasma IL-15 (pg/mL)	78.66 ± 25.43	112.78 ± 21.96	< 0.05
Plasma c-reactive protein (ng/mL)	1439.85 ± 135.94	1733.56 ± 212.38	< 0.01
Plasma p53 (pg/mL)	786.57 ± 89.32	978.96 ± 101.29	< 0.01
Plasma Bax (pg/mL)	370.86 ± 51.54	485.81 ± 62.83	< 0.01
Plasma Bcl-2 (pg/mL)	24.12 ± 4.52	15.09 ± 5.67	< 0.02
Plasma cytochrome C (pmol/mL)	78.68 ± 29.74	127.97 ± 28.49	< 0.02
Plasma caspase-3 (ng/mL)	10.32 ± 3.52	15.93 ± 5.73	< 0.01
Plasma caspase-8 (ng/mL)	63.15 ± 18.46	86.72 ± 25.37	< 0.01
Plasma caspase-9 (ng/mL)	16.24 ± 3.97	21.65 ± 4.51	< 0.05
Milk TNF-α (pg/mL)	99.35 ± 22.76	123.15 ± 26.17	< 0.02
Milk IL-2 (pg/mL)	241.63 ± 34.64	278.01 ± 29.26	< 0.01
Milk IL-10 (pg/mL)	28.94 ± 7.98	41.13 ± 10.43	< 0.01
Milk IL-12 (pg/mL)	77.87 ± 13.41	51.94 ± 17.38	< 0.02
Milk IL-15 (pg/mL)	18.24 ± 7.33	33.38 ± 9.57	< 0.05
Milk c-reactive protein (ng/mL)	896.71 ± 387.69	1463.23 ± 310.46	< 0.01
Milk p53 (pg/mL)	1089.76 ± 259.03	1286.63 ± 217.65	< 0.01
Milk Bax (pg/mL)	342.27 ± 98.70	454.61 ± 74.83	< 0.01
Milk Bcl-2 (pg/mL)	27.68 ± 8.93	13.15 ± 6.47	< 0.05
Milk cytochrome C (pmol/mL)	75.38 ± 35.74	123.96 ± 42.19	< 0.01
Milk caspase-3 (ng/mL)	19.74 ± 12.15	37.48 ± 9.77	< 0.05
Milk caspase-8 (ng/mL)	39.42 ± 18.36	61.43 ± 24.28	< 0.01
Milk caspase-9 (ng/mL)	103.14 ± 29.87	139.63 ± 17.50	< 0.01

Table S7 -Partial Pearson's correlation between the rectal temperature and candidate plasma metabolites with correction for the treatment groups (HS and HS-free).

Items	Rectal temperature
lactate	0.29*
pyruvate	0.18*
creatine	0.64*
creatinine	0.27*
proline	0.58*
lysine	0.73*
glycine	0.46*
threonine	0.37*
isoleucine	0.85*
leucine	0.66*
ornithine	0.49*
citrulline	0.83*
arginine	0.51*

*: $P < 0.01$.

Table S8 -Partial Pearson's correlation between rectal temperature and candidate milk metabolites with correction for the treatment groups (HS and HS-free).

Items	Lactate	Pyruvate	Creatine	Citrate
Rectal temperature	0.37*	0.64*	0.29*	0.78*

*: $P < 0.01$.

Table S9 -Partial Pearson's correlation between cytokine and candidate metabolites with correction of the treatment groups (HS and HS-free).

	Plasma acetone	Plasma BHBA	Milk Acetone	Milk BHBA
Plasma TNF-α	NS	NS	NS	NS
Plasma IL-2	NS	NS	NS	NS
Plasma IL-10	NS	NS	NS	NS
Plasma IL-12	NS	NS	NS	NS
Plasma IL-15	NS	NS	NS	NS
Plasma c-reactive protein	NS	NS	NS	NS
Plasma p53	0.29*	0.50*	0.43*	0.61*
Plasma Bax	0.65*	0.62*	0.45**	0.74**
Plasma Bcl-2	-0.92*	-0.31*	-0.39*	-0.63*
Plasma cytochrome C	0.58*	0.64*	0.39*	0.74*
Plasma caspase-3	NS	NS	NS	NS
Plasma caspase-8	NS	NS	NS	NS
Plasma caspase-9	NS	NS	NS	NS
Milk TNF-α	NS	NS	NS	NS
Milk IL-2	NS	NS	NS	NS
Milk IL-10	NS	NS	NS	NS
Milk IL-12	NS	NS	NS	NS
Milk IL-15	NS	NS	NS	NS
Milk c-reactive protein	NS	NS	NS	NS
Milk p53	0.37*	0.28*	0.40*	0.59*
Milk Bax	0.41*	0.74*	0.53*	0.52*
Milk Bcl-2	-0.31*	-0.71*	-0.65*	-0.52*
Milk cytochrome C	0.46*	0.43*	0.29*	0.54*
Milk caspase-3	NS	NS	NS	NS
Milk caspase-8	NS	NS	NS	NS
Milk caspase-9	NS	NS	NS	NS

*: $P < 0.01$. **: $P < 0.001$. NS, no significant correlations.

Table S10 -Partial Pearson's correlation between inflammation markers and candidate metabolites with correction of the treatment groups (HS and HS-free).

	Milk cytochrome C	Milk c-reactive protein
Plasma cytochrome C	0.71**	0.32*
Plasma c-reactive protein	0.47*	0.79**

*: $P < 0.01$. **: $P < 0.001$
